# TBProfiler for automated calling of the association with drug resistance of variants in *Mycobacterium tuberculosis*

**DOI:** 10.1371/journal.pone.0279644

**Published:** 2022-12-30

**Authors:** Lennert Verboven, Jody Phelan, Tim H. Heupink, Annelies Van Rie

**Affiliations:** 1 Torch Consortium FAMPOP Faculty of Medicine and Health Sciences, University of Antwerp, Antwerp, Belgium; 2 ADReM Data Lab, Department of Computer Science, University of Antwerp, Antwerp, Belgium; 3 Faculty of Infectious and Tropical Diseases, Department of Infection Biology, London School of Hygiene and Tropical Medicine, London, United Kingdom; Newcastle University, UK, UNITED KINGDOM

## Abstract

Following a huge global effort, the first World Health Organization (WHO)-endorsed catalogue of 17,356 variants in the *Mycobacterium tuberculosis* complex along with their classification as associated with resistance (interim), not associated with resistance (interim) or uncertain significance was made public In June 2021. This marks a critical step towards the application of next generation sequencing (NGS) data for clinical care. Unfortunately, the variant format used makes it difficult to look up variants when NGS data is generated by other bioinformatics pipelines. Furthermore, the large number of variants of uncertain significance in the catalogue hamper its useability in clinical practice. We successfully converted 98.3% of variants from the WHO catalogue format to the standardized HGVS format. We also created TBProfiler version 4.4.0 to automate the calling of all variants located in the tier 1 and 2 candidate resistance genes along with their classification when listed in the WHO catalogue. Using a representative sample of 339 clinical isolates from South Africa containing 691 variants in a tier 1 or 2 gene, TBProfiler classified 105 (15%) variants as conferring resistance, 72 (10%) as not conferring resistance and 514 (74%) as unclassified, with an average of 29 unclassified variants per isolate. Using a second cohort of 56 clinical isolates from a TB outbreak in Spain containing 21 variants in the tier 1 and 2 genes, TBProfiler classified 13 (61.9%) as unclassified, 7 (33.3%) as not conferring resistance, and a single variant (4.8%) classified as conferring resistance. Continued global efforts using standardized methods for genotyping, phenotyping and bioinformatic analyses will be essential to ensure that knowledge on genomic variants translates into improved patient care.

## Introduction

Tuberculosis (TB) remains an important public health problem with 10 million new cases each year of which about 500,000 cases are rifampicin resistant tuberculosis [1]. Genomic drug resistance testing (gDST) by whole genome sequencing (WGS) or targeted next generation sequencing (tNGS) can be used to determine the resistance profile of *Mycobacterium tuberculosis* (*Mtb*) strains.

In June 2021 the WHO endorsed the first catalogue of mutations in *Mtb* complex and their association with drug resistance [2]. To create the WHO catalogue, 41 countries contributed data of five or more isolates, 32 countries on more than 50 isolates, and 17 on more than 500 isolates. The prevalence of resistance in the WHO dataset ranged from 0.6% for clofazimine toe 40.5% for ethionamide. A list of tier 1 and tier 2 candidate resistance genes was selected (Table 1) and paired WGS and phenotype data from over 38,000 *Mtb* isolates was analyzed to determine the odds ratios (ORs) for the association with resistance of each variant in the candidate resistance genes. Based on the ORs, variants were classified as “associated with resistance”, “not associated with resistance” or “uncertain significance”. In addition, an interim category was used for “associated with resistance” and “not associated with resistance” to reflect uncertainty in some observed associations.

**Table 1:** The tier 1 and 2 genes from the WHO catalogue [2].

Drug	Tier 1	Tier 2
**Isoniazid**	*ahpC*, *inhA*, *katG*	*mshA*, *ndh*, *Rv1258c*, *Rv2752c*
**Rifampicin**	*rpoB*	*rpoA*, *rpoC*, *Rv2752c*
**Ethambutol**	*embA*, *embB*, *embC*	*embR*, *ubiA*
**Pyrazinamide**	*pncA*, *clpC1*, *panD*	*Rv1258c*, *PPE35*, *Rv3236c*
**Fluoroquinolones**	*gyrA*, *gyrB*	
**Bedaquiline**	*pepQ*, *Rv0678*, *mmpL5*, *mmpS5*, *atpE*	*Rv1979c*
**Linezolid**	*rplC*, *rrl*	
**Clofazimine**	*pepQ*, *Rv0678*, *mmpL5*, *mmpS5*	*Rv1979c*
**Delamanid**	*fgd1*, *ddn*, *fbiA*, *fbiB*, *fbiC*, *Rv2983*	
**Amikacin**	*rrs*, *eis*, *whiB7*	*whiB6*, *ccsA*, *fprA*, *aftB*
**Streptomycin**	*rrs*, *rpsL*, *gid*, *whiB7*, *Rv1258c*	*whiB6*
**Ethionamide**	*inhA*, *ethA*	*ethR*, *mshA*, *Rv3083*, *ndh*
**Kanamycin**	*rrs*, *eis*, *whiB7*	
**Capreomycin**	*rrs*, *tlyA*	*whiB6*, *ccsA*, *fprA*, *aftB*

The information in the catalogue greatly advances our knowledge on genomic causes of resistance in *Mtb*, which increases our ability to predict clinically relevant resistance phenotypes from genetic data. Unfortunately, the format in which the variants are presented in the WHO catalogue is not user-friendly. The Clockwork bioinformatics pipeline [3] combined with the piezo software [4] used to analyze the raw sequencing data presents variants in a format that differs from the format used by most *Mtb* bioinformatics pipelines (such as PhyResSE [5], MTBSeq [6], TBProfiler [7] and XBS [8]). This makes it difficult for researchers and clinicians to efficiently use the WHO catalogue or to look up the classification of a variant identified in an *Mtb* isolate when using other bioinformatics pipelines.

In this study, we aimed to standardize the notation of *Mtb* variant reporting and automate the calling of variants in the tier 1 and tier 2 candidate resistance genes. To standardize the variant notation, we developed a method to convert how *Mtb* variants are listed in the WHO catalogue to the Human Genome Variation Society (HGVS) sequence variant nomenclature format [9]. The HGVS sequence variant nomenclature was chosen because provides a consistent and unambiguous description of variants, is compliant with the “Nomenclature for Incompletely Specified Bases in Nucleic Acid Sequence” [10], is commissioned by a working group of three international organizations (Human Genome Variation Society, Human Variome Project, and the Human Genome Organization), is widely adopted and is acknowledged as the standards nomenclature in molecular diagnostics [11–13]. This ensures that the description of all sequence variants is standardized and adheres to recommendations for the reporting of sequence variants in a clinical setting [14]. To facilitate automation, we created a new version of TBProfiler [7] to call variants in candidate resistance genes from raw read files or VCF files [15] generated by WGS or tNGS pipelines and to classify the variants identified as “associated with resistance”, “not associated with resistance” or “unclassified” based on the 2021 WHO catalogue of mutations. To evaluate the use of the new version of TBProfiler, we applied the tool for resistance calling of WGS data obtained from clinical *Mtb* isolates collected from a cohort of 340 South African patients diagnosed with rifampicin resistant TB.

## Methods

### Conversion from the WHO catalogue format to HGVS notation

Genomic variants in tier 1 and tier 2 *Mtb* candidate resistance genes can occur in regions that code for rRNA, regions that code for amino acids, or promotor regions of coding regions. Variants can be single nucleotide polymorphisms (SNPs), multiple nucleotide polymorphisms (MNPs), insertions or deletions. SNPs and MNPs in the coding region can be classified as synonymous if the variant does not result in a change in amino acid, or missense (also called non-synonymous) if the variant results in a change at amino acid level. For protein coding genes, missense variants should be presented at the amino-acid level. Variants that occur in promoter regions or in genes that code for rRNA do not translate to amino acids and can thus not be classified as synonymous or non-synonymous. For these genes, variants should be presented at the nucleotide level. Insertions and deletions in all genes and all promoter regions should also presented at nucleotide level.

To convert the annotation of variants from the WHO catalogue notation to the standard HGVS notation, we used regular expressions (regex) and re-ordered the information captured by the regex (S1 Table). The code to translate the WHO catalogue is publicly accessible from ‘https://github.com/LennertVerboven/WHO_catalogue_paper’.

Missense variants in genes coding for amino acids are listed in the catalogue as ‘gene_XNY’, where X is the single letter amino acid code for the reference allele (for example A for alanine), Y the single letter amino acid code for the alternative allele and N the codon in the gene where the variant occurs. By using a regex and translating the single letter amino acid code to the three letter abbreviations, ‘gene_XNY’ can be transformed to the HGVS format gene_p.abcNdef. For example, *rpoB*_S450L is converted to *rpoB*_p.Ser450Leu.

Variants in the promoter region of a candidate resistance gene are presented in the catalogue as ‘gene_xNy’ where ‘gene’ is the candidate resistance gene in which the variant occurs, ‘x’ is the reference allele, ‘y’ the alternative allele, and ‘N’ is a negative number indicating the location of the variant, i.e., the number of bases before the start of the coding region of the gene where the variant is located. By using a regex and reordering the information, the variant ‘gene_xNy’ can be transformed to the HGVS notation ‘gene c.Nx>y’. While the HGVS specifications recommend that promoter variants are reported based on their location within the reference genome, we opted to list promoter variants based on their relative position to the coding gene, as this is common practice in the *Mtb* sequencing community. For example, *embA*_c-12t listed was converted to *embA*_c.-12c>t.

Variants in genes coding for rRNA (for example *rrl* and *rrs*) are reported in the catalogue at the nucleotide level as ‘gene_xNy’. These variants were transformed to the HGVS format in a similar way as variants in promoter regions. For example, ‘rrs_a1401g’ becomes *rrs*_n.1401a>g. Note the difference in gene_c.Nx>y for coding regions for genes and gene_n.Nx>y for variants in genes coding for rRNA. Throughout all conversions at nucleotide level (i.e., insertions, deletions, promotor variants, and variants in rRNA) the distinction between regions coding for genes and regions coding for rRNA is made.

**Fig 1:**
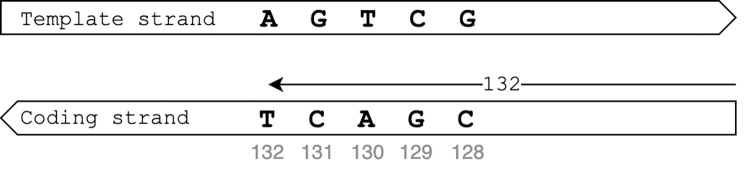
Conversion of the variant position for genes on the template strand using the reference allele for whiB6_132_agtcg as example.

**Fig 2:**
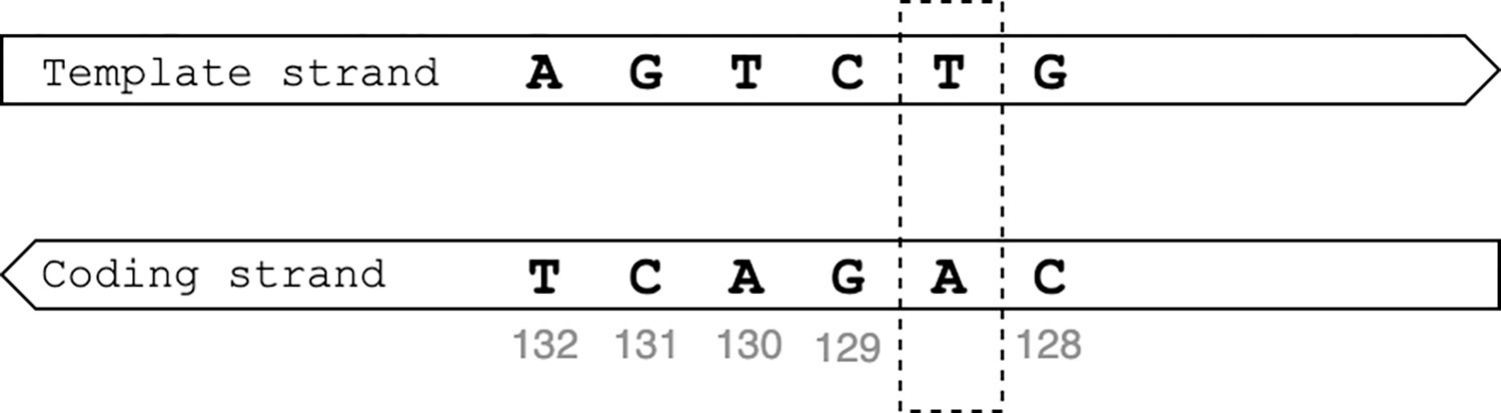
Conversion of the variant position for genes on the template strand using the insertion whiB6_132_ins_1_agtcg_agtctg as example.

The catalogue lists insertions as ‘gene_N_ins_L_x_y’ where gene represents the gene where the insertion occurs, N the start position of the first base in the reference allele, L the length of the insertion, x the reference allele at nucleotide level and y the alternative allele at nucleotide level. The location ‘N’ only indicates the first position of the reference allele. For the HGVS notation, the locations of both nucleotides that flank the inserted nucleotides are required. For genes on the template strand of the DNA, the start location of the insertion was determined by aligning the reference and alternative alleles with a gap of length L in the reference allele. The positions of the HGVS flanking nucleotides were then computed by taking the location of the left and right flanking nucleotides of the gap in the alignment and adding those to the value for N. For the example, for insertion ‘*rrs*_88_ins_1_gatac_gatact’ the left flanking nucleotide is at position 92 (insertion ‘t’ occurs after nucleotide ‘c’ which lies in position 92 i.e., the 4^th^ position after ‘g’ in position 88) and the right flanking nucleotide is 93 (92 plus 1). Insertion ‘*rrs*_88_ins_1_gatac_gatact’ is thus translated to ‘*rrs*_n.92_93insT’. For genes on the coding strand, the catalogue reports the variant position on the coding strand and the nucleotides on the template strand, whereas the HGVS notation reports both the variant position and the nucleotides on the coding strand. To generate the HGVS notation, the nucleotides first had to be complemented to represent the nucleotides on the coding strand and the order of the bases had to be reversed to match the direction of the coding strand as shown in Fig 1. For example, ‘*whiB6*_132_ins_1_agtcg_agtctg’ means that the reference allele `agtcg`is located from position 132 to 128 on the coding strand and a nucleotide ‘t’ is inserted is between ‘c’ and ‘g’ on the template strand (Fig 2). From the perspective of the coding strand, the position remains 132 to 128 but a single nucleotide ‘a’ is inserted between nucleotide ‘g’ and ‘c’. The position of the left flanking nucleotide is then calculated as ‘N’ minus ‘length of allele’ plus ‘position of the nucleotide after which the insertion occurs’ (132–5+1 = 128 in the example). The position of the right flanking nucleotide is the position of the left flanking nucleotide plus 1 (128+1 = 129 in the example). The insertion ‘*whiB6*_132_ins_1_agtcg_agtctg’ was thus converted to ‘*whiB6*_c.128_129insA’.

Some insertions listed as unique in the catalogue can be located at multiple locations in the gene. For example, the single nucleotide ‘c’ insertion in ‘*rrs*_1108_ins_1_gtctcat_gtctccat’ could have been inserted between ‘t’ in position 1111 and ‘c’ in position 1112, or between ‘c’ in position 1112 and ‘a’ in position 1113. The variant normalization rules [9] state that an insertion or deletion should be left aligned, meaning that the start position should be shifted as far to the left as possible. For ‘rrs_1108_ins_1_gtctcat_gtctccat’, the correct transformation would thus be *rrs*_n.1111_1112insC. To ensure compatibility with commonly used *Mtb* pipelines, we opted to list all possibilities. For example, when converting ‘*rrs*_1108_ins_1_gtctcat_gtctccat’ to HGVS format, both *rrs*_n.1111_1112insC and *rrs*_n.1112_1113insC were generated.

**Fig 3:**
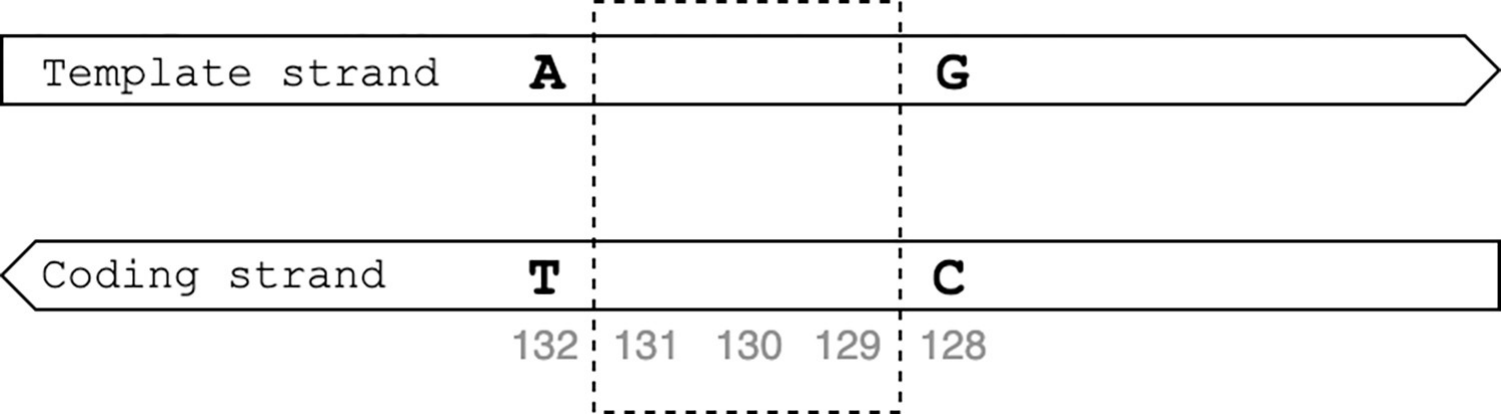
Conversion of the variant position for genes on the template strand using the deletion whiB6_132_del_3_agtcg_ag as example.

Deletions follow the same structure as insertions and are represented as gene_N_del_L_x_y, where gene represents the gene name, N is the start position of the reference allele, L is the length of the deletion, and x and y are the reference and alternative alleles. Conversion of deletions from the WHO catalogue to HGVS format was performed in a similar way as for insertions, with the exception that only the position of the flanking bases is required, and the deleted nucleotides are not reported. For genes on the template strand, the reference and alternative allele were aligned to determine which base(s) in the reference allele were deleted and the position thereof. For example, ‘rpoB_1308_del_3_gaac_g’ is converted to rpoB_c.1309_1311del as the deletion starts one nucleotide after nucleotide ‘g’ in position 1308 and the deletion is 3 nucleotides long. For genes transcribed from the coding strand, the start location of the deletion had to be subtracted from the start position in the WHO catalogue notation. For example, the start position of the deletion ‘*whiB6*_132_del_3_agtcg_ag’ is determined as ‘N’ minus ‘length of allele’ plus ‘position of first deleted base’ (132–5+2 = 129 in the example); the end position is calculated as ‘N’ minus ‘length of allele’ plus ‘position of last deleted nucleotide as compared to the first deleted nucleotide’ (132–5+4 = 131) (Fig 3). The variant ‘*whiB6*_132_del_3_agtcg_ag’ is thus converted to ‘*whiB6*_c.129_131del’. Like insertions, there might be multiple HGVS notations that represent a unique deletion in the WHO catalogue notation. For example, the nine bases deleted in ‘*rpoB*_1293_del_9_ccaattcatgga_cca’, can either be aattcatgg when the deletion starts at first ‘a’ (‘ccaattcatga’) or can be attcatgga if the deletion starts at the second (‘ccaattcatgga’) ‘a’. The variant ‘*rpoB*_1293_del_9_ccaattcatgga_cca’, thus results in HGVS notations *rpoB*_c.1295_1303del and *rpoB*_c.1296_1304del.

When variants lie the coding region of one gene and the promoter region of another gene (which occurred 1,626 times in the WHO catalogue), the catalogue reports the variant deemed most important in the context of drug resistance and places the other in between brackets. For example, variant *inhA*_g-154a (*fabG1*_L203L) lies in the promoter region of the *inhA* gene and in the coding region of the *fabG1* gene. Because the *inhA*_g-154a variant is more likely to cause the association with resistance to isoniazid than the synonymous L203L mutation in the *fabG1* gene, the variant is reported as *inhA*_g-154a (*fabG1*_L203L). Because the statistics to determine the association with resistance in the WHO catalogue were only estimated for the variant not in brackets, *inhA*_g-154a (*fabG1*_L203L) was converted to HGVS notation *inhA*_c.-154g>a. When the annotation listed between brackets fall in a promoter region or results in an amino acid change, it is more difficult to identify with confidence which of the two variants may confer resistance. In such case, we still only converted the variant placed not in brackets because the statistics used to determine the association with resistance does not apply to the other variant. For example, *inhA*_c.c-522g (*fabG1*_p.Pro81Ala) is converted to *inhA*_c.-522c>g.

### Development of TBProfiler version 4.4.0

TBProfiler version 4.4.0 was developed to automate drug resistance calling using the information published in the WHO catalogue of mutations in *Mtb* complex. After generating the HGVS notation for all variants included in the 2021 WHO catalogue, the new notation was used to create a ‘HGVS WHO catalogue’ database which contains all variants in HGVS notation (plus original WHO catalogue notation for reference) and their association with resistance. In addition, TBProfiler 4.4.0 lists all variants in the tier 1 and 2 genes detected in NGS data even those that were not listed in the 2021 WHO catalogue.

TBProfiler 4.4.0 can be used with one of two default variant databases or with any custom-made database. In the ‘2021 WHO TBProfiler database’, the classification of a variant is listed as ‘associated with resistance’ or ‘not associated with resistance’ solely based on the information contained in the WHO catalogue. In the ‘TBDB TBProfiler database’, the information on association with resistance contained in the WHO catalogue is complemented with a curated list of variants [16].

When loading one of the two default or a custom-made variant database, TBProfiler v4.4.0 extracts the genomic position in the H37Rv reference genome together with the reference and alternate alleles and their confidence grading classification from the variant database and stores them in VCF format. The VCF file is then annotated in HGVS format with SnpEff [17] for functional annotation of variants. All variants with their functional annotation and confidence grading are then stored. When analyzing samples (either from raw fastq data or VCF files), TBProfiler then performs a direct lookup of the variant in the database that was loaded. Using SnpEff when loading a database and analyzing a sample ensures that variants that are functionally equal and will match between the samples and the database. All variants present in the sample that are present in the database and classified as ‘associated with resistance’ and ‘not associated with resistance’ will be reported as such and all variants in the tier 1 and 2 genes not listed in the database are listed as unclassified variants. Variants with multiple functional annotations, such as the *inhA*_c.-154g>a variant which also causes the synonymous *fabG1*_p.Leu203Leu variant have their second annotation (in this case *fabG1*_p.Leu203Leu) listed as additional information when reporting the *inhA*_c.-154g>a variant.

### Analysis of WGS data from clinical *Mtb* isolates using TBProfiler 4.4.0

We integrated TB profiler V4.4.0. with the ‘2021 WHO TBProfiler database’ for drug resistance variant calling in the XBS bioinformatics pipeline to analyze data from 340 clinical isolates from a representative cohort of patients diagnosed with rifampicin resistant TB in three provinces (Eastern Cape, Free State, and Gauteng) of South Africa diagnosed in 2012 or 2013 (ENA accession number PRJEB57919) during the EXIT-RIF study [18,19]. In addition, we analyzed a dataset of 59 isolates obtained from a TB outbreak which occurred in Aragon, Spain in 2020 (ENA accession number PRJNA781095) to assess the useability of the WHO catalogue for surveillance. For these analyses, we used the TBProfiler output to estimate the prevalence of variants ‘associated with resistance’, ‘not associated with resistance’ or ‘unclassified’ variants’ overall and for individual drugs. We also estimated the number of unclassified variants by isolate, overall and for those not yet seen in the NGS data of a previously processed isolate from the same patient cohort.

## Results

### Conversion of WHO catalogue to HGVS notation

Of the 17,356 variants listed in the WHO catalogue, 17,061 (98.3%) could be converted to the standard HGVS notation. Of the 295 variants (30 insertions and 265 deletions) that could not be converted, most (n = 258, 87.5%) were classified as ‘uncertain significance’, some (n = 33, 11.2%) as ‘associated with resistance interim’, and few (n = 4, 1.4%) as ‘not associated with resistance’.

Most (16,992 or 97.9%) variants could be converted using the 4 regular expressions (regexes) listed in S1 Table. Of the remaining 364 (2.1%) that failed to convert using the regexes, 110 (98 deletions and 12 insertions) had a length mismatch between the reference, alternative allele, and the length of the indel due to truncation of the reference and alternative alleles at a length of 50 bases by the piezo software [4], and 254 had multiple variants grouped as a single indel, something the HGVS notation does not allow unless the multiple variants lie in the same codon. For the indels with length mismatch, we could manually impute the missing bases using the H37Rv reference genome for 69 of the 98 deletions. The remaining 29 deletions could not be converted because they contained multiple variants grouped into a single indel. The mismatch in 12 insertions were also unsolvable as the contents of the insertion could not be assumed. The 254 cases where Clockwork had grouped multiple variants as a single indel could in principle be split manually, but the derived indels can then not be classified as ‘associated with resistance’, ‘not associated with resistance’ or ‘unknown significance’ because the statistics used for the WHO catalogue were performed for the multiple indels together. For example, in ‘*whiB6*_-73_del_1_agctctagtg_agtctagta’ the first ‘c’ is deleted, but the last base also changed from a ‘g’ to an ‘a’. Manually splitting these indels and converting to *whiB6*_c.71del and *whib6*_c.64g>a is possible but these variants cannot be correctly classified.

### TBProfiler version 4.4.0 analysis of *Mtb* WGS data

#### South African cohort of rifampicin resistant isolates

**Fig 4:**
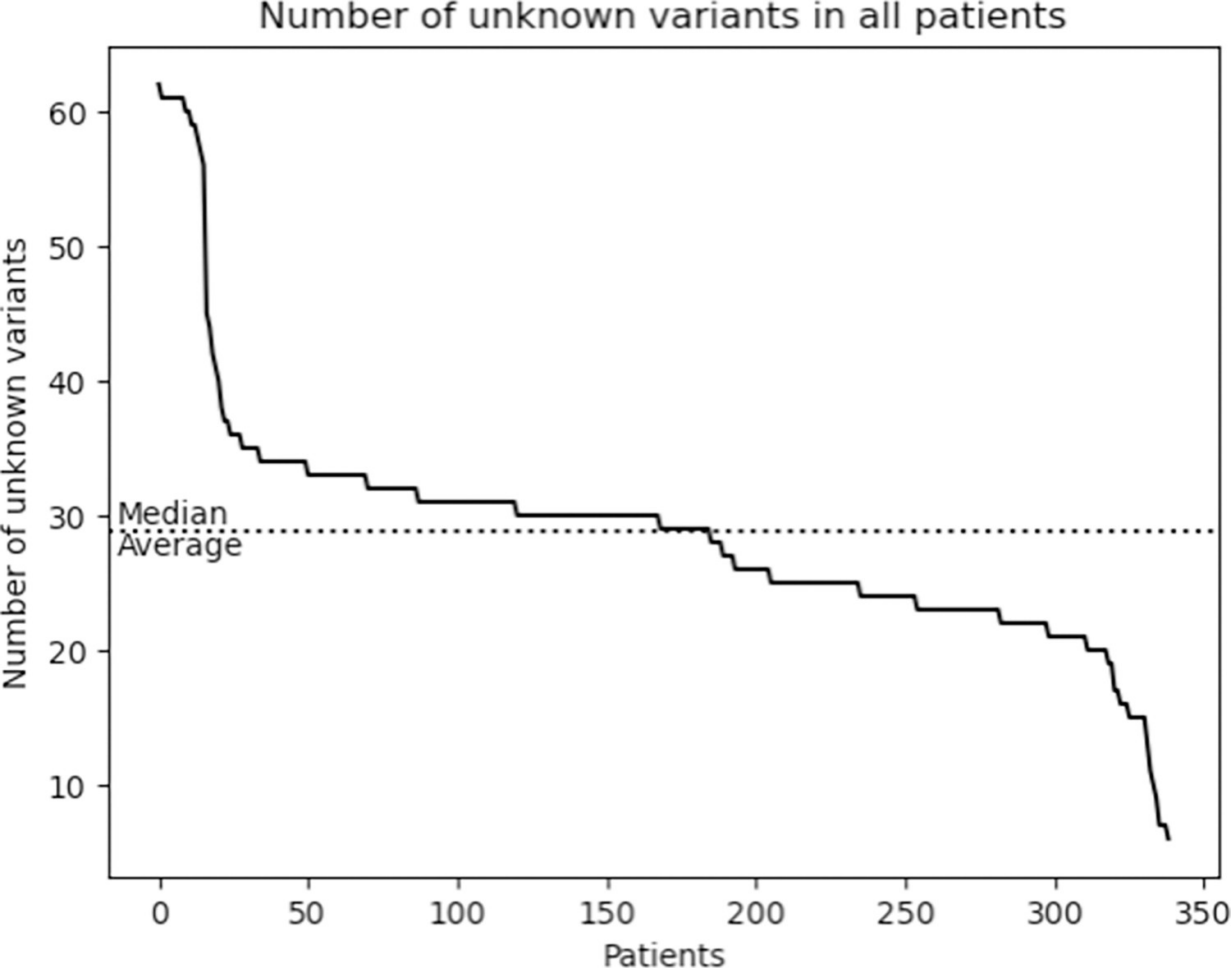
Number of unclassified variants in tier 1 or 2 genes (unknown association with drug resistance) according to the WHO catalogue in WGS data of 339 clinical Mtb isolates.

**Fig 5:**
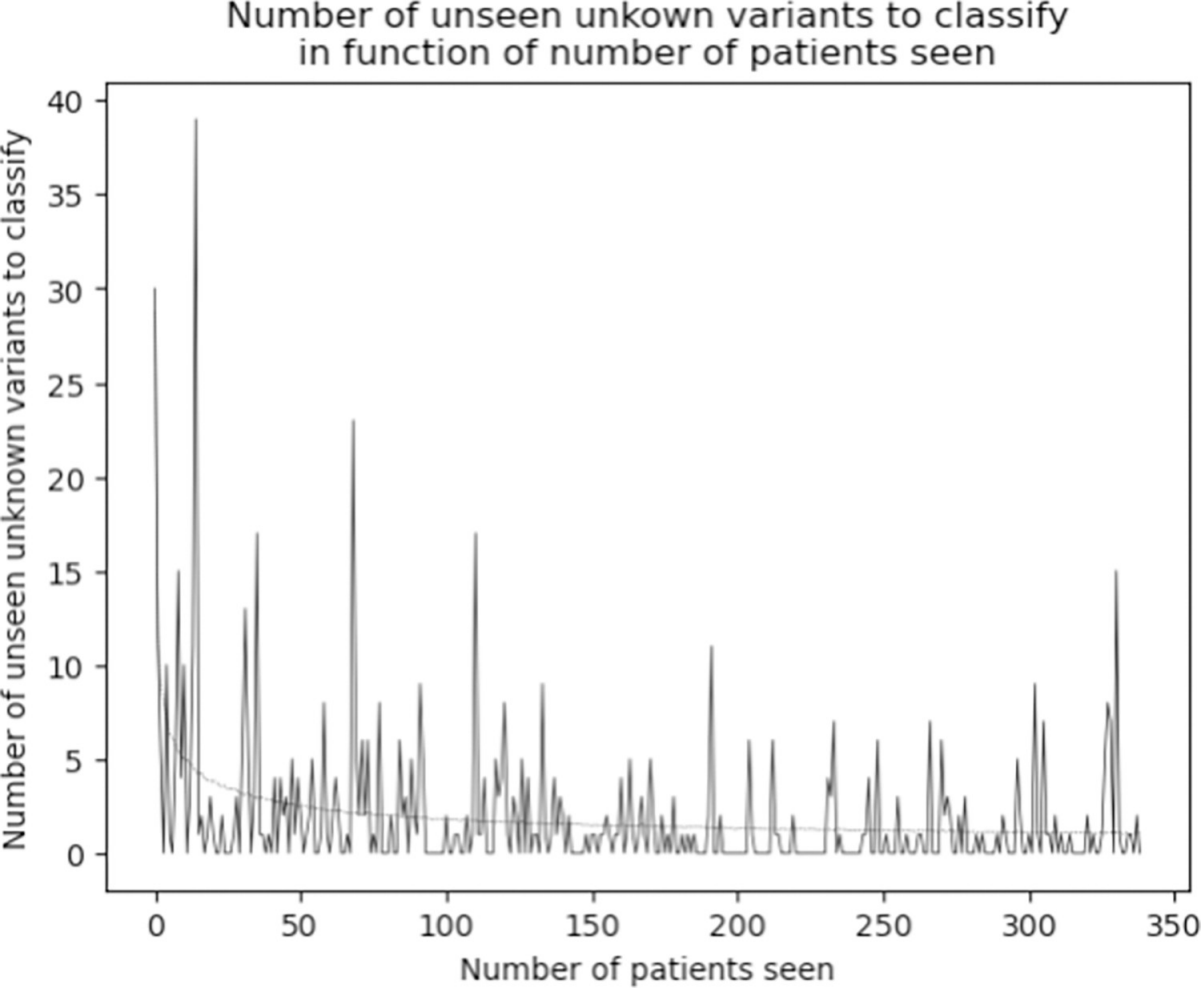
Number of unseen unknown variants in a new patient in function of the number of patients previously seen.

In the 340 *Mtb* culture isolates, a total of 812 variants were identified in the tier 1 and 2 candidate resistance genes. One isolate with 121 unique variants, of which 79 were located in the highly conserved *rrs* and *rrl* genes, was excluded as these variants were most likely due to contamination. After removal of the contaminated isolate, 691 unique variants in the tier 1 and 2 genes remained in the analysis. Of these 691 variants in the remaining 339 isolates, 105 (15.2%) were classified as ‘associated with resistance’, 72 (10.4%) as ‘not associated with resistance’, and 514 (74.4%) as ‘unclassified’ meaning that they either had the ‘uncertain significance’ classification in the WHO catalogue, or were not present at all in the catalogue. The median number of variants in tier 1 or 2 genes with unclassified association to drug resistance was 29 per *Mtb* isolate and ranged from 6 to 62 (Fig 4). When analyzing the 339 isolates sequentially, 174 isolates contained at least one unclassified variant that had not yet been seen in a previous isolate from the same clinical cohort. The number of unseen unclassified variants was very high for the first few patients after which it decreased rapidly but peaks of over 10 unclassified variants occurred even after NGS data of 250 isolates had been analyzed (Fig 5).

**Fig 6:**
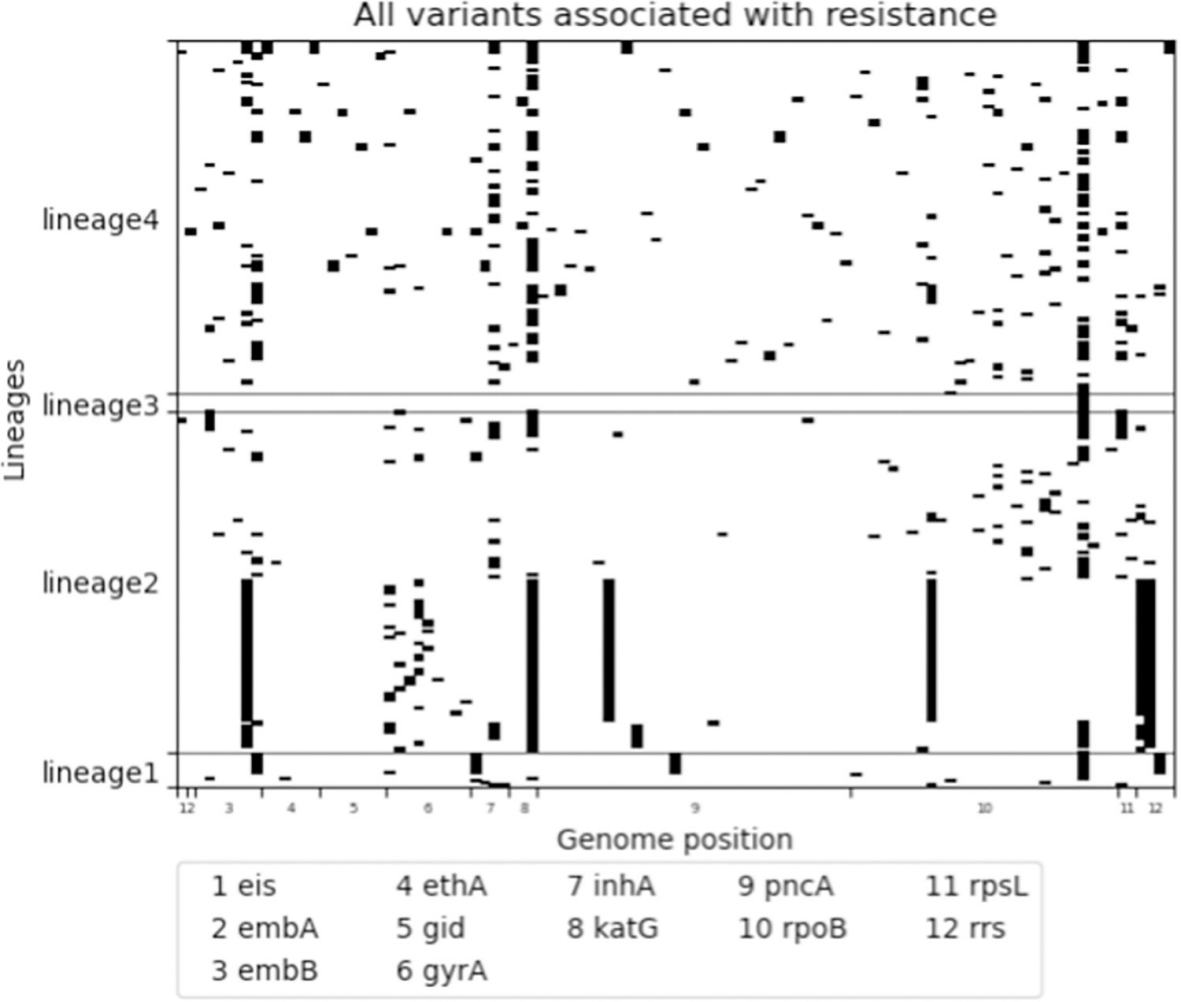
105 variants annotated and classified as associated with resistance by TBProfiler 4.4.0. Data is presented by lineage (x-axis) and genome position (y-axis). Data is presented by lineage (x-axis) and genome position (y-axis). Variants were identified in WGS dataset of 339 clinical Mtb isolates from South African patients diagnosed with rifampicin resistant tuberculosis.

**Fig 7:**
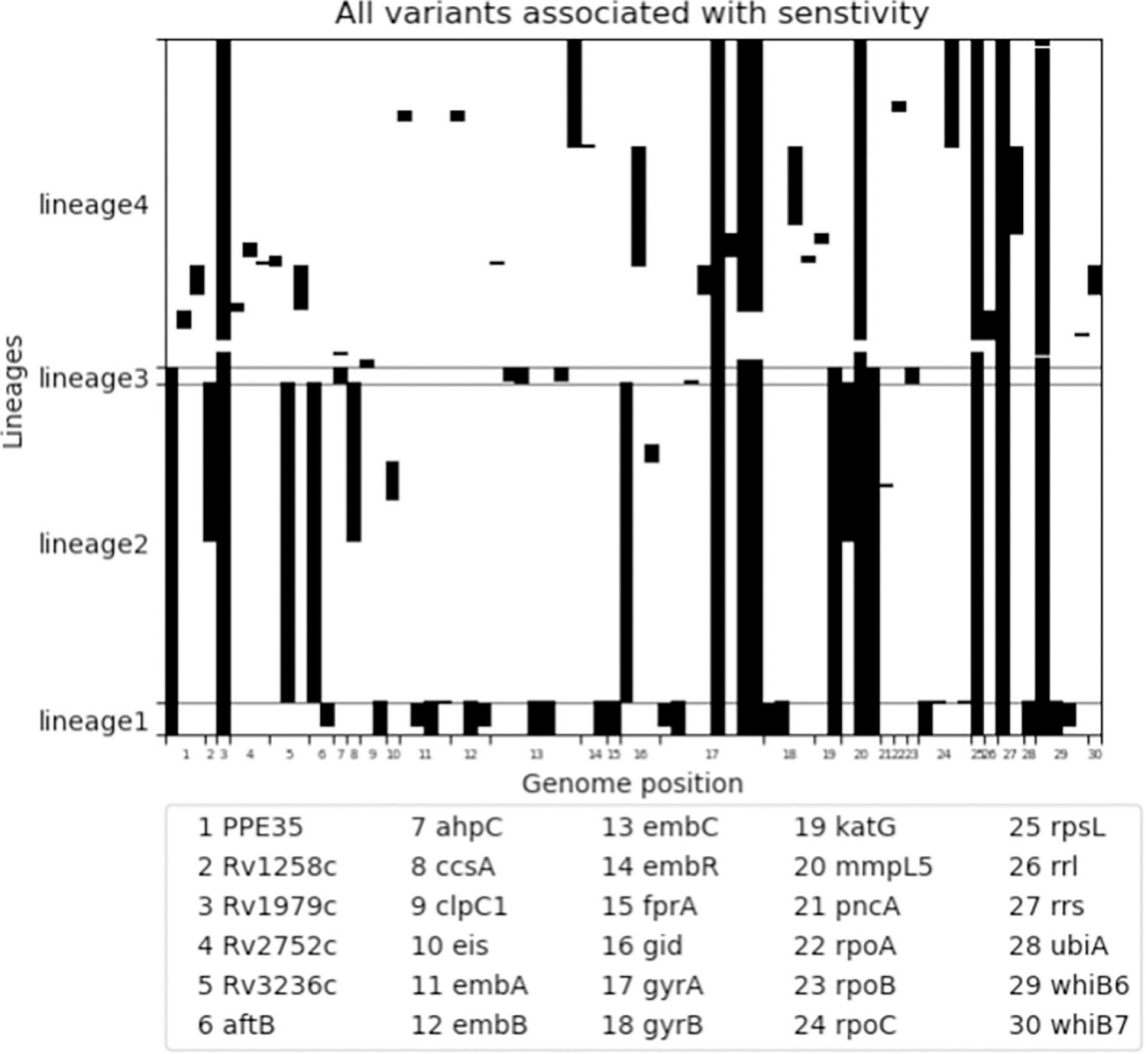
71 variants annotated and classified as ‘not associated with resistance’ by TBProfiler 4.4.0. Data is presented by lineage (x-axis) and genome position (y-axis). Data is presented by lineage (x-axis) and genome position (y-axis). Variants were identified in WGS dataset of 339 clinical Mtb isolates from South African patients diagnosed with rifampicin resistant tuberculosis.

**Fig 8:**
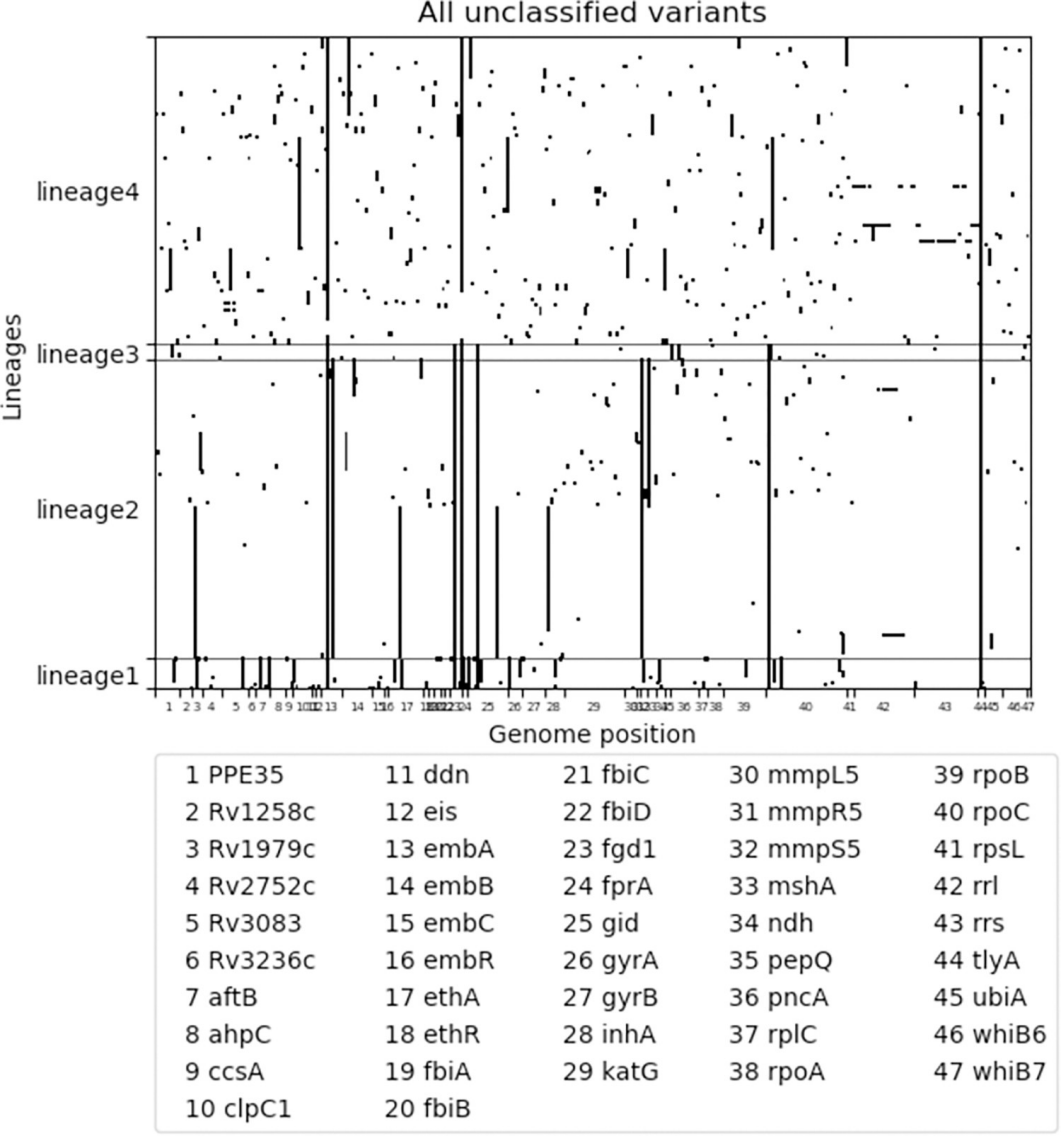
636 variants annotated by TBProfiler 4.4.0. that could not be classified (variant of unknown significance in WHO catalogue, or variant not listed in WHO catalogue). Data is presented by lineage (x-axis) and genome position (y-axis). Data is presented by lineage (x-axis) and genome position (y-axis). Variants were identified in WGS dataset of 339 clinical Mtb isolates from South African patients diagnosed with rifampicin resistant tuberculosis.

Variants ‘associated with resistance’ were mainly homoplastic with independent occurrence in multiple lineages (Fig 6) and occur sporadically in all lineages. Some variants associated with resistance were due to the spread of a clonal strain, as for example the large cluster of a lineage 2 strain (Fig 6). The variants classified as ‘not associated with resistance’ were predominantly (sub-)lineage markers that occurred in large monophyletic clusters with only a few variants occurring in single isolates (Fig 7). The unclassified variants (i.e., variants classified as ‘unknown significance’ in WHO catalogue, and variants not listed in the WHO catalogue) were a mix of homoplastic variants occurring independently in multiple lineages and monophyletic variants occurring in large clades spanning several (sub-)lineages (Fig 8). Fifteen monophyletic unclassified variants occurred in more than 50 clinical isolates (Table 2). These variants included six synonymous variants which were by rule excluded from the WHO catalogue page 61 [2], four promotor variants that lie far upstream of the coding gene (-779 to -339), and four missense variants. The variants spanning the largest monophyletic clade were the synonymous variants occurring in 300 out of the 339 isolates.

**Table 2:** All unclassified variants occurring in more than 50 isolates.

Variant	Number of occurrences	Drugs and their classification	Likely reason
***tlyA*_c.33A>G**	339	Capreomycin: not listed	Synonymous variant
***embA*_c.-590C>T**	330	Ethambutol: not listed	Far upstream
***fprA*_c.-11_-10insA**	315	Capreomycin: not listed Amikacin: not listed	
***gid*_c.615A>G**	179	Streptomycin: not listed	Synonymous variant
***fgd1*_c.960T>C**	179	Delamanid: not listed	Synonymous variant
***rpoC*_c.-339T>C**	179	Rifampicin: not listed	Far upstream
***mmpS5*_c.-710C>G**	155	Bedaquiline: not listed	Far upstream
***embA*_c.228C>T**	155	Ethambutol: not listed	Synonymous variant
***Rv1979c*_p.Arg409Gln**	78	Bedaquiline: uncertain Clofazimine: uncertain	
***gid*_p.Leu79Ser**	78	Streptomycin: uncertain	
***ethA*_p.Ala381Pro**	78	Ethionamide: uncertain	
***mshA*_p.Ala187Val**	77	Isoniazid: Not associated Ethionamide: Uncertain	
***inhA*_c.-779G>T**	64	Isoniazid: uncertain Ethionamide: uncertain	Far upstream
***clpC1*_c.2418C>T**	58	Pyrazinamide: not listed	Synonymous variant
***rpoC*_c.1626C>G**	58	Rifampicin: not listed	Synonymous variant

The distribution by category differed by drug (Table 3). While on average, 15.2% of variants in tier 1 or 2 genes were classified as ‘associated with resistance’, this proportion what highest (>20%) for rifampicin, pyrazinamide, and fluoroquinolones and lowest (0%) for the new and reproposed drugs bedaquiline, clofazimine, linezolid and delamanid. The proportion of variants in tier 1 and 2 genes classified as ‘not associated with resistance’ was highest (>20%) for ethambutol and fluoroquinolones and lowest (≤1%) for linezolid, delamanid and ethionamide. The proportion of unclassified variants was high (>50%) for all drugs and reached ≥99% for linezolid and delamanid.

**Table 3:** Distribution overall and by drug of WHO classification of 691 unique variants in tier 1 and tier 2 genes identified by WGS in 339 clinical isolates of patients diagnosed in South Africa with rifampicin resistant TB.

	Number of unique variants	Associated with resistance	Not associated with resistance	Unclassified*
	691 (100%)	105 (15.2%)	71 (10.3%)	515 (74.5%)
**First line drugs**				
**Rifampicin**	130	28 (21.5%)	9 (9.6%)	93 (71.5%)
**Isoniazid**	99	6 (6.1%)	11 (11.1%)	82 (82.8%)
**Pyrazinamide**	96	33 (34.4%)	10 (10.4%)	53 (55.2%)
**Ethambutol**	75	8 (10.7%)	17 (22.7%)	50 (66.7%)
**RR-TB drugs—WHO Group A**				
**Levofloxacin**	43	9 (20.9%)	12 (27.9%)	22 (51.2%)
**Moxifloxacin**	43	9 (20.9%)	10 (23.3%)	24 (55.8%)
**Bedaquiline**	26	0 (0.0%)	4 (15.4%)	22 (84.6%)
**Linezolid**	81	0 (0.0%)	2 (2.5%)	79 (97.5%)
**RR-TB drugs—WHO Group B**				
**Clofazimine**	27	0 (0.0%)	5 (19.2%)	21 (80.8%)
**RR-TB drugs—WHO Group C**				
**Delamanid**	21	0 (0.0%)	0 (0.0%)	21 (100.0%)
**Amikacin**	87	2 (2.3%)	12 (13.8%)	73 (83.9%)
**Ethionamide/Prothionamide**	72	10 (13.9%)	0 (0.0%)	62 (86.1%)
**Other TB drugs**				
**Capreomycin**	83	1 (1.2%)	10 (12.0%)	72 (86.7%)
**Kanamycin**	48	2 (4.2%)	4 (8.3%)	42 (87.5%)
**Streptomycin**	112	12 (10.7%)	9 (8.0%)	91 (81.2%)

#### Isolates from a TB outbreak in Aragon Spain

From the 57 isolates retrieved from patients involved in a TB outbreak in Aragon, Spain, 56 passed WGS quality control. In these 56 isolates, a total of 19 variants were found in the tier 1 and 2 candidate drug resistance genes. Of these 21 variants, one (4.8%) variant (gyrA Asp94Gly) present in a single isolate was classified as ‘associated with resistance’ to fluoroquinolones, seven (33.3%) were classified as ‘not associated with resistance’ with six of these seven variants being present in all samples and the remaining variant present in all samples but one, and 13 (61.9%) variants were unclassified, meaning they were not represented in the catalogue, or were listed as being of ‘uncertain association’, with a median of seven unclassified variants per isolates.

## Discussion

Genomic DST (gDST) by NGS, including WGS and targeted deep sequencing, could become a revolutionary tool for the control of drug resistant TB as it allows for rapid detection of the complete resistance phenotype [20–22]. To date, NGS has been endorsed for surveillance but not yet for clinical care [23]. To increase the efficacy of the use of NGS in research and surveillance and to enable the use of NGS for clinical care, standardization and automation are essential [24].

The publication of the WHO endorsed catalogue of mutations in *Mtb* and their association with resistance [2] contains crucial data for the development of novel rapid molecular tests and for the integration of NGS-based gDST into clinical practice. Especially for indels, looking up variants encountered in clinical or research isolates in the WHO catalogue is difficult due to the use of a non-standard format for listing the variants. In this study, we successfully converted the WHO catalogue notation of 98.3% of the 17,356 variants listed to their HGVS notation, which follows a published standard [14]. This benefits the users when looking up a variant encountered in NGS data and could result in more homogeneity in reporting variants in publications.

To further facilitate the use of NGS data, we integrated the information present in WHO catalogue into a new version of the TBProfiler, a tool commonly used by bioinformatics pipelines for resistance calling of *Mtb* variants. TBProfiler version 4.4.0 automatically calls all variants in any tier 1 or 2 candidate resistance gene and classifies them as ‘associated with resistance, ‘not associated with resistance’ or ‘unclassified’. The latter includes variants of unknown significance in the WHO catalogue and variants that not included in the 2021 version of the WHO catalogue.

To assess the application of the new TBProfiler 4.4.0 for the drug resistance calling of WGS data of *Mtb* isolates, we purposefully selected two distinct sets of isolates: a cohort of 339 South African patients with rifampicin resistant TB and 56 patients involved in a TB outbreak in Spain. The representative sample of rifampicin resistant TB cases in South Africa can give a good indication of the useability of the WHO catalogue for the management of drug resistant tuberculosis, while the Spanish dataset can shed light on the useability of the WHO catalogue in an outbreak setting. As expected, a higher proportion of variants classified as ‘associated with resistance’ were observed in the rifampicin resistance cohort as compared to the outbreak cohort (105/691 or 15.2% versus 1/21 or 4.8%), and a lower proportion of variants classified as ‘not associated with resistance’ were recorded in the rifampicin resistant isolates as compared to the outbreak isolates (72/691 or 10.4% versus 7/21 or 33.3%). In both groups, the majority of variants observed were either classified in the WHO catalogue as ‘of unknown significance’ or were not listed in the WHO catalogue (514/691 or 74.4% of the rifampicin resistant isolates and 13/21 or 61.9% of the outbreak isolates). Similarly, 82% of the 17,356 variants listed in the WHO catalogue were classified as “of unknown significance” [2], This high proportion complicates the clinical use of the NGS data as resolution of ‘unclassified’ variants requires literature review and/or expert consultation to determine the most likely association with resistance of these variants.

Our study has several limitations. First our study is based on one representative cohort of clinical rifampicin resistant Mtb isolates from the three provinces in South Africa and one outbreak in Spain. While we observed a high proportion of unclassified variants in both settings, the exact proportion of unclassified variants may differ by geographic region. Second, even though we were able to convert most variants in the catalogue, 33 variants ‘associated with interim resistance’ and 4 variants ‘not associated with resistance’ could not be converted to the standard HGVS format. The exclusion of these variants might have slightly biased our estimate of the proportion of variants in different categories. Third, because the NGS data were not used for clinical care, a comprehensive review of the literature or expert consultation was not done for the 514 unique unclassified variants. As such, the importance of classifying these variants cannot be determined based on the data presented.

In conclusion, the WHO catalogue is a large stride towards improved management of rifampicin resistant TB using WGS or tNGS. In this study, we removed some barriers for use of WGS for clinical care by improving standardization of variant reporting and automation of variant calling. To fully implement WGS or tNGS in clinical care, continued global efforts will be needed to reduce the number of unclassified variants. This may require an open-access system dedicated to the collection and review of variants encountered in clinical isolates that are of ‘uncertain significance’ and variants not yet listed that are. In future, data compendia and catalogues should be published in a standardized format such as the HGVS.

## Supporting information

S1 Table Conversion of variants in the WHO catalogue format used in the WHO catalogue to the standard HGVS format: Generic regular expressions by variant type and examples. (PDF)

S1 File (CSV)

S2 File (CSV)
