# Correction: TBProfiler for automated calling of the association with drug resistance of variants in *Mycobacterium tuberculosis*

**DOI:** 10.1371/journal.pone.0293254

**Published:** 2023-10-17

**Authors:** Lennert Verboven, Jody Phelan, Tim H. Heupink, Annelies Van Rie

There are errors in the Funding section. The correct Funding statement is: LV, 1SB4519N, Fonds Wetenschappelijk Onderzoek, https://www.fwo.be/. AVR, G0F8316N, Fonds Wetenschappelijk Onderzoek, https://www.fwo.be/. AVR, T001018N, Fonds Wetenschappelijk Onderzoek, https://www.fwo.be/.

## References

[1] Verboven L, Phelan J, Heupink TH, Van Rie A (2022) TBProfiler for automated calling of the association with drug resistance of variants in *Mycobacterium tuberculosis*. PLoS ONE 17(12): e0279644. 10.1371/journal.pone.0279644 36584023 PMC9803136

