# Supplementary material for: TBProfiler for automated calling of the association with drug resistance of variants in Mycobacterium tuberculosis
Source: PLoS One. 2022 Dec 30;17(12):e0279644. doi: 10.1371/journal.pone.0279644 (PMC9803136; doi:10.1371/journal.pone.0279644)
Supplement: S1 Table — (PDF) [file pone.0279644.s001.pdf]

Type		WHO catalogue	Regex	HGVS
**missense variant in AA coding gene**	generic	gene_XNY	^(\w+)_([A-Z])(\d+)([A-Z!])$	gene_p.XNY
	example	rpoB_S450L	^(rpoB)_(S)(450)(L)$	rpoB_p.Ser450Leu
**variant in promotor region**	generic	gene_xNy	^(\w+)_([actg])(-*\d+)([actg])$	gene_c.Nx>y
	example	embA_c-12t	^(embA)_(c)(-12)(t)$	embA_c.-12c>t
**variant in rRNA coding gene**	generic	gene_xNy	^(\w+)_([actg])(-*\d+)([actg])$	gene_n.Nx>y
	example	rrs_a1401g	^(rrs)_(a)(1401)(g)$	rrs_n.1401a>g
**insertion**	generic	gene_N_ins_L_x_y	^(\w+)_(-*\d+)_ins_(\d+)_([actg]+)_([actg]+)$	
**insertion in gene on coding strand**	example	rrs_88_ins_1_gatac_gatact	^(rrs)_(88)_ins_(1)_(gatac)_(gatact)	rrs_ n.92_93insT
**insertion in gene on template strand**	example	whiB6_321_ins_1_gcgc_gccgc	^(whiB6)_(321)_ins_(1)_(gcgc)_(gccgc)$	whiB6_c.320_321insG
**insertion with multiple options**	example	rrs_1108_ins_1_gtctcat_gtctccat	^(rrs)_(1108)_ins_(1)_(gtctcat)_(gtctccat)$	rrs_n.1111_1112insC
				rrs_n.1112_1113insC
**deletion**	generic	gene_N_del_L_x_y	^(\w+)_(-*\d+)_del_(\d+)_([actg]+)_([actg]+)$	
**deletion in gene on coding strand**	example	rpoB_1308_del_3_gaac_g	^(rpoB)_(1308)_del_(3)_(gaac)_(g)	rpoB_c.1309_1311del
**deletion in gene on template strand**	example	whiB6_132_del_3_agtc_a	^(whiB6)_(132)_del_(3)_(agtc)_(a)$	whiB6_c.129_131del
**deletion with multiple options**	example	rpoB_1293_del_9_ccaattcatgga_cca	^(rpoB)_(1293)_del_(9)_(ccaattcatgga)_(cca)$	rpoB_c.1295_1303del
				rpoB_c.1296_1304del
**Multiple variants**	example	inhA_c.c-522g (fabG1_p.Pro81Ala)		inhA_c.-522c>g

S1 Table
